# Influence of visual objects and music on anxiety levels and imaging process in patients undergoing coronary CT angiography

**DOI:** 10.1007/s00330-025-11614-0

**Published:** 2025-04-24

**Authors:** Muhammed Tekinhatun, Kadir Han Alver, İbrahim Akbudak, Mehmet Turmak, Eyyup Çavdar, Muhammed Akif Deniz

**Affiliations:** 1 Department of Radiology, Dicle University, Diyarbakir, Turkey; 2 Department of Radiology, Pamukkale University, Denizli, Turkey; 3 Department of Medical Oncology, Tekirdağ Namık Kemal University, Tekirdağ, Turkey

**Keywords:** Anxiety, Music, Computed tomography angiography, Visual perception, Waiting rooms

## Abstract

**Objective:**

High anxiety during coronary computed tomography angiography (CCTA) can compromise imaging quality, increase radiation exposure, and elevate medication use. Therefore, optimizing waiting room environments to reduce patient anxiety is important for clinical outcomes. This study examines the effects of music and visual stimuli in the waiting rooms on patients’ anxiety levels, heart rate, radiation dose, and beta-blocker use prior to CCTA.

**Methods:**

This study, designed as a prospective and randomized trial, was conducted between April 15 and August 15, 2024, with 216 patients randomized into two groups: a standard waiting room (SWR) and a designed waiting room (DWR) featuring music and visual objects. Anxiety and depression levels were measured using the Hospital Anxiety and Depression Scale (HADS) and the State-Trait Anxiety Inventory (STAI). Additional parameters, such as heart rate, radiation dose, and beta-blocker requirement, were also recorded.

**Results:**

In the DWR group, anxiety scores and heart rates were significantly lower compared to the SWR group (*p* < 0.001). Additionally, a notable reduction in radiation dose and beta-blocker use was observed in the DWR group (*p* < 0.05). In the general patient population, higher anxiety scores were associated with poorer imaging quality. Imaging quality was significantly better in the DWR group (*p* < 0.001).

**Conclusion:**

It has been demonstrated that waiting room designs enriched with music and visual stimuli reduce anxiety during CCTA scanning, enhancing patient comfort, improving imaging quality, and enabling imaging with lower radiation doses. The design of such waiting rooms can improve patient experience while optimizing outcomes.

**Key Points:**

***Question*** *Can a waiting room with music and visual stimuli reduce anxiety and heart rate in CCTA patients, improving imaging quality and reducing beta-blocker use?*

***Findings*** *Music and visual stimuli reduced anxiety and heart rate, lowering beta-blocker use and radiation doses while improving imaging quality in CCTA*.

***Clinical relevance*** *Integrating music and visual stimuli in waiting rooms helps reduce anxiety and heart rate, leading to less medication use and radiation exposure while enhancing imaging quality. This simple, cost-effective approach improves patient comfort and optimizes outcomes in CCTA procedures*.

**Graphical Abstract:**

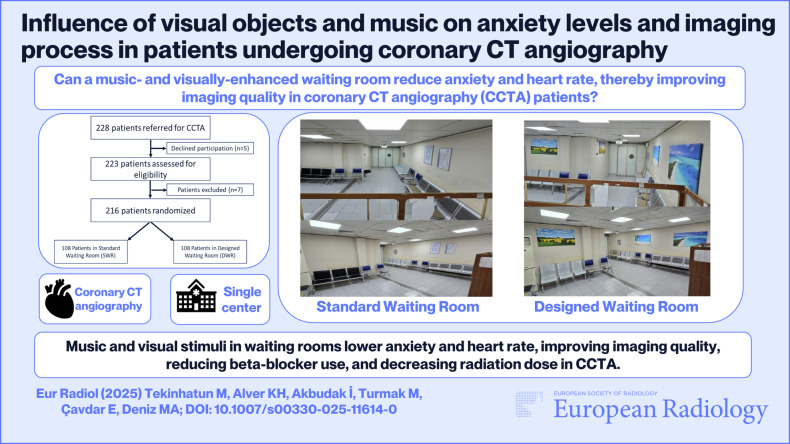

## Introduction

Anxiety, first defined by Sigmund Freud, is a physiological response that includes feelings of fear, worry, unease, and irritability, which arise when a person feels insecure in the face of an undefined danger or unknown threat [1, 2]. In a state of anxiety, the individual experiences a sense of alarm and anticipates the emergence of a negative outcome. Such emotional states disrupt daily activities and reduce the quality of life [1–3].

Studies indicate that nearly half of outpatients undergoing medical imaging procedures experience high levels of anxiety, with the highest levels observed in MRI, followed by CT procedures [4–10]. Among patients undergoing coronary CT angiography (CCTA), anxiety is often elevated due to a lack of understanding of the imaging process, the critical importance of results, and concerns about the potential treatment following diagnosis. Pre-CCTA anxiety has been reported in 47.3–74.1% of patients and is associated with increased heart and respiratory rates, poorer image quality, higher beta-blocker use, and suboptimal imaging outcomes [8, 11–16].

Cardiovascular diseases, particularly coronary artery disease (CAD), are leading causes of mortality globally [17]. Accurate diagnosis and effective treatment planning are essential, as even patients with non-obstructive CAD face a significantly elevated risk of major adverse cardiovascular events (MACE) (pooled OR, 3.17, 95% CI: 2.77–3.63) [18]. For anatomic assessment of CAD, traditional invasive coronary angiography is now complemented by noninvasive CCTA, supported by robust clinical evidence and multinational guidelines [19]. The growing utilization of CCTA as a non-invasive imaging technique has revealed a prevalence of non-obstructive CAD ranging from 15% to 37%, underlining the need for high-quality imaging [18]. Despite its advantages, CCTA imaging poses challenges such as respiratory motion, heart rate fluctuations, arrhythmias, and psychological factors like patient anxiety. Providing a suitable imaging environment to reduce anxiety not only mitigates these challenges but also enhances diagnostic accuracy and patient outcomes [17, 18].

Non-pharmacological interventions, such as music and visual stimuli, are increasingly used in hospital environments for their stress-reducing benefits [2, 20, 21]. Research suggests that these interventions, combined with well-ventilated and spacious waiting rooms, can significantly lower patient anxiety [22–29]. However, their specific impact on patients undergoing CCTA remains underexplored. This study aims to evaluate how visual stimuli and music in a designed waiting room (DWR) affect anxiety scores, heart rates, radiation doses, and beta-blocker usage, compared to a standard waiting room (SWR), ultimately aiming to enhance image quality and patient comfort.

## Materials and methods

### Participants, study design, and procedure

Patients referred to our department for CCTA between April 15, 2024, and August 15, 2024, were included in this study, which was conducted in accordance with the 1995 Helsinki Declaration. The study commenced after obtaining local ethics committee approval on February 14, 2024 (decision number 125). Patients aged 18 years or older referred to our unit for CCTA were eligible for inclusion.

**Fig. 1:**
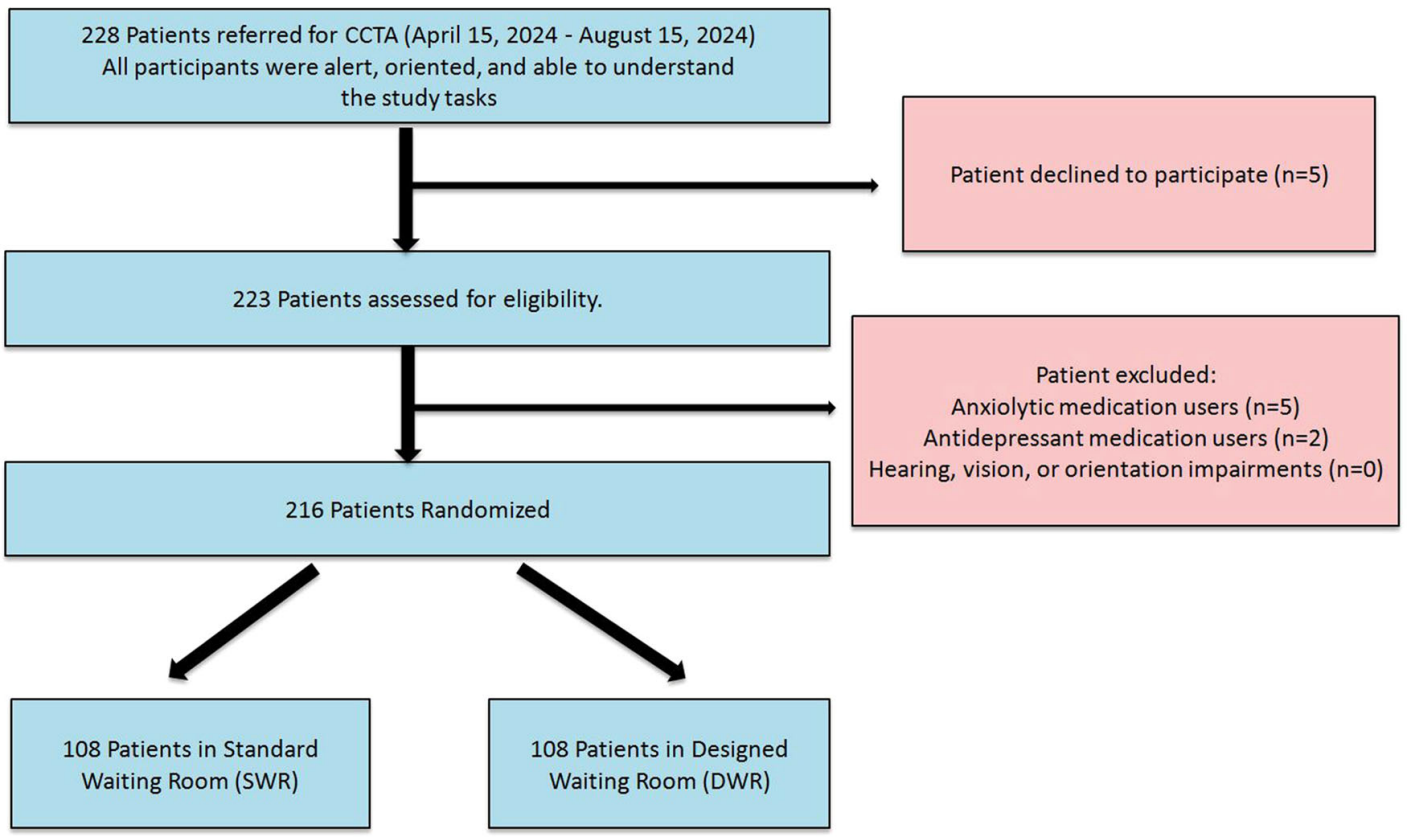
Flowchart of patients undergoing CCTA. CCTA, coronary computed tomography angiography; SWR, standard waiting room; DWR, designed waiting room

A total of 228 patients were initially assessed for eligibility. Exclusion criteria included hearing, vision, or orientation impairments, and the use of anxiolytics or antidepressants. However, no patients met the exclusion criteria related to hearing, vision, or orientation. All participants were alert, oriented, and able to understand the study tasks. Five patients declined to participate, and seven were excluded due to medication use (anxiolytics: *n* = 5, antidepressants: *n* = 2). Thus, 216 patients meeting the inclusion criteria were randomized for the study (Fig. 1).

**Fig. 2:**
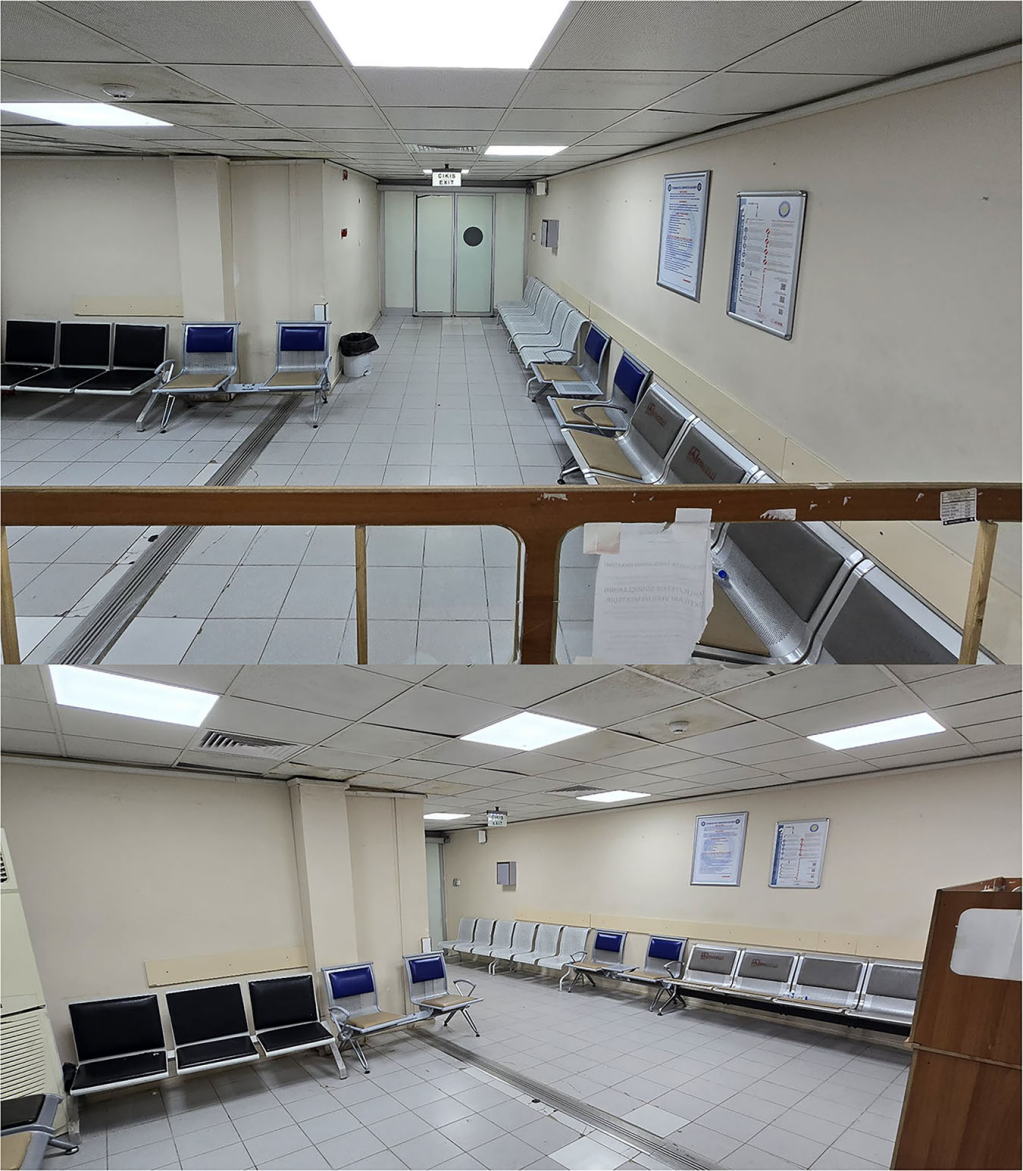
The appearance of the SWR

**Fig. 3:**
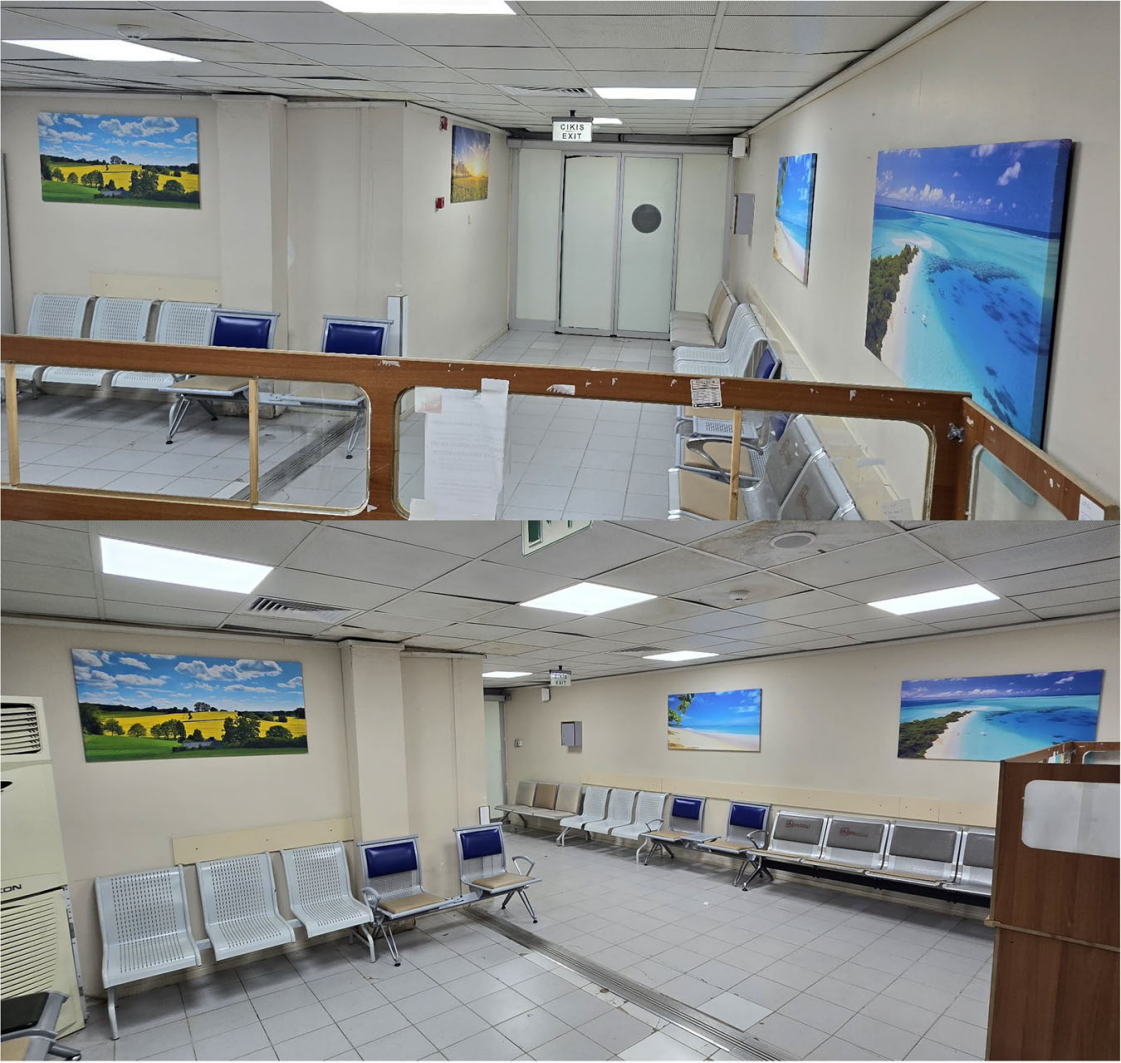
The appearance of the DWR, featuring calming visual elements and ambient music to create a relaxing environment for patients awaiting CCTA. CCTA, coronary computed tomography angiography

Our study was designed as a prospective, randomized, controlled trial. All cardiac imaging procedures were performed on a separate device during regular working hours. The same waiting room was alternately arranged each week as either a SWR (Fig. 2) or a DWR featuring music and visual objects (Fig. 3). Patients scheduled for CCTA scans were sequentially assigned to the setup in place for that week. A total of 216 patients were randomized, with 108 patients in each group.

The time spent in the hospital was recorded when patients arrived for CCTA scanning. Patients were seated in the waiting rooms for a 10-min relaxation period, after which informed consent was obtained in person, and their heart rates were measured by nurses. They then completed the Hospital Anxiety and Depression Scale (HADS) and the State-Trait Anxiety Inventory (STAI) forms. Following these procedures, patients were directed to the relevant department for imaging. Additionally, body mass index (BMI), educational level, marital status, and monthly income were recorded. Before imaging, another heart rate measurement was taken; scans were performed for patients with a heart rate < 65 bpm. For those with a heart rate > 65 bpm, a beta-blocker (metoprolol 50–100 mg) was administered based on cardiology recommendation [30]. CCTA was initiated 45–60 min after the first dose of the beta-blocker, once an appropriate heart rate was achieved. If necessary, additional doses were given, with the total beta-blocker dose reaching up to 200 mg [30–34].

Routine CCTA examinations in our clinic are performed using a dual-source CT scanner with 128 × 2 slices and two X-ray tubes at a 95° angle (Somatom Definition Flash, Siemens Healthcare). Non-contrast images with 3-mm slice thickness were acquired for calcium scoring (Ca score) using the Agatston method. Contrast-enhanced scans, performed with electrocardiogram (ECG)-gating, covered the area from the carina to the heart’s diaphragmatic surface. Three imaging protocols were used based on heart rate and arrhythmia: Flash mode for heart rates < 65 bpm without arrhythmias, Adaptive mode for 65–90 bpm or < 65 bpm with arrhythmias, and Spiral mode for heart rates > 90 bpm. ECG-gated protocols ensured tailored imaging, and heart rate was measured before but not after the scan.

### CCTA image quality assessment

In the evaluation of coronary arteries, a four-point scale was used for CCTA images of vessels with a diameter ≥ 1.5 mm: Absence of motion artifacts and clear vessel outlines (excellent), minor artifacts with slight blurring (good), blurring artifacts without disruption of the vessel course (adequate), severe artifacts causing doubling or disruption of the vessel course (inadequate) [14, 35].

Grade 3 was considered acceptable image quality for routine clinical practice. Two experienced radiologists evaluated the consensus image quality using a four-point qualitative scale that categorized the images as excellent, good, adequate, or inadequate [14, 35].

The differences in heart rate, anxiety scores, and image quality between males and females were analyzed in both the general population and in the DWR and SWR groups. The frequencies within each group were reported separately to compare image quality using the qualitative scale.

### DWR

Speakers mounted on the ceiling were connected to a computer, ensuring equal distribution of music throughout the room. The track “3 h of Relaxing Guitar Music: Meditation Music, Instrumental Music, Calming Music, Soft Music,” which has proven effective in previous studies, was played for free via YouTube at youtube.be/ss7EJPW2Uk.

Four images with dominant shades of blue and green were downloaded for free from pixabay.com (ID numbers: 3369304, 2135026, 1993704, and 2413078) to promote a sense of relaxation. These images were professionally printed on 120 × 70 cm cotton canvases. The paintings were mounted on walls, each illuminated by spotlights and positioned equidistantly from viewing perspectives to ensure full visibility.

### The HADS

The HADS is a reliable assessment tool developed to measure anxiety and depression levels in outpatient populations. Created by Zigmond and Snaith in 1983, the scale consists of 14 items divided into two subscales: HADS-Anxiety (7 items) and HADS-Depression (7 items) [36]. Each item is scored from 0 to 3, with each subscale yielding a minimum score of 0 and a maximum score of 21. Higher scores are positively correlated with anxiety and depression levels. The Turkish version of the scale, translated by Aydemir and colleagues, has been reported to be a valid and reliable tool for the Turkish population [2]. The collected forms were later evaluated by a specialist psychologist following standardized protocols, without direct patient interaction or clinical interpretation.

### The STAI

The STAI, developed by Spielberger, is a reliable questionnaire known for measuring state and trait anxiety and consists of two sections, each with 20 questions. The first section, STAI-State Anxiety, assesses participants’ feelings ‘at the moment,’ while the second section, STAI-Trait Anxiety, evaluates how they generally feel. Responses to each question are scored between one and four based on symptom severity. Each subscale is evaluated separately, with scores ranging from twenty to eighty; higher scores indicate higher anxiety levels. The STAI was translated into Turkish and validated by Öner and LeCompte [2].

### Patient dose

The scanning protocols and doses received by each patient were evaluated individually. For each CCTA, the CT dose index volume (CTDIvol) and dose-length product (DLP) were recorded. The DLP (unit: mGy•cm) represents the average dose absorbed by the patient per sequence, calculated by multiplying the CTDIvol by the scan length. Real-time CTDIvol and DLP displays on the CT monitor were collected and analyzed via the picture archiving and communication system (PACS). The effective radiation dose (ED) was calculated in millisieverts (mSv) by multiplying the DLP by the organ dose coefficient of 0.014 mSv/mGy•cm.

### Power analysis

In a similar study using an anxiety scale, the mean anxiety scores between groups were found to be 9.0 ± 4.5 in the SWR group and 6.9 ± 3.6 in the DWR group [2]. Based on these data, a power analysis was conducted, yielding a required sample size of 198 for 95% power with a 5% margin of error. Our study included a total of 216 patients, with 108 in the control group and 108 in the study group.

### Statistical analyses

Descriptive statistics were summarized as frequency and percentage for categorical variables, and as mean and standard deviation for continuous variables. The Kolmogorov–Smirnov test was used to assess the normality of the data distribution. For independent and continuous variables, the independent samples *t*-test was employed for normally distributed data, while the Mann–Whitney *U*-test was used for non-normally distributed data. Chi-square tests were applied for categorical variable comparisons, and Fisher’s exact test was used when the chi-square test was not suitable. Correlations between variables were analyzed using Pearson correlation for normally distributed data and Spearman correlation for non-normally distributed data. Statistical analyses were conducted using SPSS software version 25.0 (IBM SPSS Statistics for Windows, IBM Corp.), and a *p*-value < 0.05 was considered statistically significant.

## Results

### Patient characteristics

After applying the exclusion criteria, 216 patients who were alert, oriented, and able to fully participate in the study were included. A total of 12 patients were excluded due to medication use or refusal to participate. These patients were then randomized into the SWR or DWR groups. All measurements were conducted in person. The mean age of the participants was 49.2 ± 11.8 years, with a median age of 49 years (range: 18–77). The majority of the patients were male (65.7%), and 85.2% were married. No statistically significant differences were observed between the SWR and DWR groups in terms of age, BMI, gender distribution, marital status, income levels, or educational status (*p* > 0.05) (Table 1).

**Table 1:** Patient characteristics and demographics

	All patients (*n* = 216)	SWR group (108)	DWR group (108)	*p*-value
Age (median)	49	49	49.50	0.3585
BMI (kg/m^2^)	28.40	27.68	29.64	0.0652
Gender				
• Male	142 (65.74%)	74 (68.52%)	68 (62.96%)	0.4735
• Female	74 (34.26%)	34 (31.48%)	40 (37.04%)	
Marital status				
• Single	32 (14.81%)	17 (15.74%)	15 (13.89%)	0.8481
• Married	184 (85.19%)	91 (84.26%)	93 (86.11%)	
Monthly income (USD)^*^				
• 300–600	111 (51.39%)	58 (53.70%)	53 (49.07%)	0.1498
• 600–1000	49 (22.69%)	28 (25.93%)	21 (19.44%)	
• 1000 and above	56 (25.93%)	22 (20.37%)	34 (31.48%)	
Education status				
• Elementary school	99 (45.83%)	53 (49.07%)	46 (42.59%)	0.1463
• High school	54 (25.00%)	30 (27.78%)	24 (22.22%)	
• College or higher	63 (29.17%)	25 (23.15%)	38 (35.19%)	

^*^ In our country, the net minimum wage is approximately 500 USD

*SWR* standard waiting room, *DWR* designed waiting room, *BMI* body mass index

### Comparative analysis of SWR and DWR groups

Patients in the DWR waited an average of 23.8 min (range: 12–50), while those in the SWR waited an average of 23.1 min (range: 14–53), with no significant difference between the groups (*p* = 0.340). However, patients in the DWR group demonstrated a significantly lower need for beta-blocker usage and a lower ED (*p* < 0.001 and *p* = 0.001, respectively) (Table 2).

**Table 2:** Comparison of measurement results and other characteristics of patients in the standard and design waiting rooms

		SWR group (*n* = 108)		DWR group (*n* = 108)		
		Mean ± std/*n*	Median	Mean ± std/*n*	Median	*p*-value
Beta blocker use	Yes	50 (46.3%)		23 (21.3%)		< 0.001
	No	58 (53.7%)		85 (78.7%)		
Effective dose (mSv)		11.4 ± 5.3	12.3	9 ± 4.4	9.8	0.001
Wait time (min)		23.8 ± 0.7	22	23.1 ± 0.8	20.5	0.340
HADS	Anxiety	8.5 ± 0.4	8.5	6.0 ± 0.4	6	< 0.001
	Depression	7.0 ± 0.3	7.0	6.4 ± 0.4	6	0.156
STAI	State Anxiety	39.6 ± 0.8	40.0	37.3 ± 0.8	37.5	0.046
	Trait Anxiety	43.5 ± 0.7	43.0	42.8 ± 0.8	43.5	0.708
Pulse rate (per min)		76.8 ± 1.2	76.5	71.0 ± 1.0	69.5	< 0.001

*SWR* standard waiting room, *DWR* designed waiting room, *HADS* hospital anxiety and depression scale, *STAI* state-trait anxiety inventory

The mean HADS-anxiety score in the SWR group was 8.5 ± 0.4, compared to 6.0 ± 0.4 in the DWR group (*p* < 0.001). The mean STAI-state anxiety score was 39.6 ± 0.8 in the SWR group and 37.3 ± 0.8 in the DWR group (*p* = 0.046). No significant differences were found between the groups for HADS-depression scores or STAI-trait anxiety scores (*p* = 0.156 and *p* = 0.708, respectively). The mean pulse rate of patients in the SWR group was 76.8 beats per minute, while it was 71.0 beats per minute in the DWR group, a statistically significant difference (*p* < 0.001) (Table 2).

**Fig. 4:**
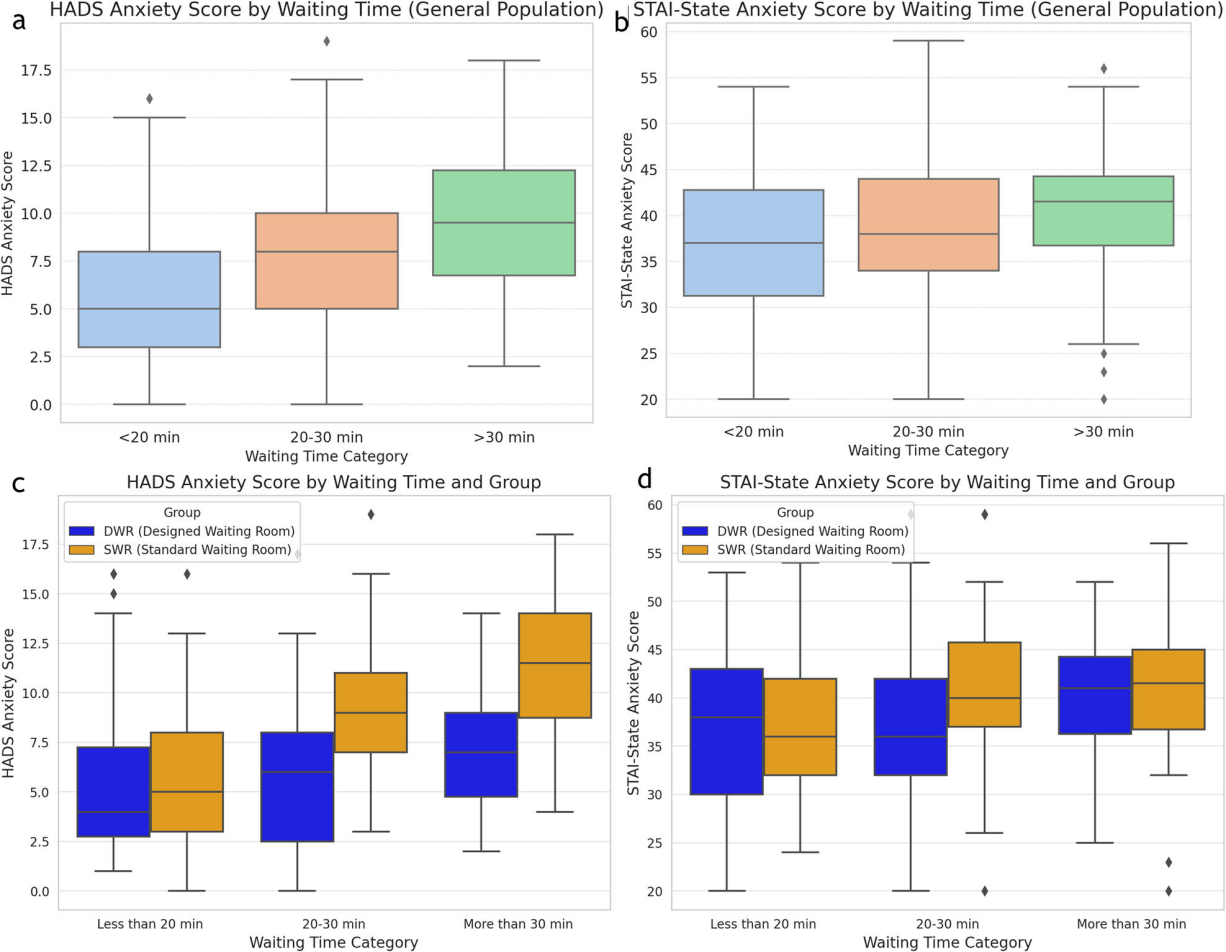
Box plots illustrating the distribution of HADS-anxiety scores and STAI-state anxiety scores across different waiting time categories. The first two figures represent the general patient population (**a**, **b**), while the latter two figures (**c**, **d**) compare the SWR and DWR groups. HADS, hospital anxiety and depression scale; STAI, state-trait anxiety inventory; SWR, standard waiting room; DWR, designed waiting room

For all patients, anxiety levels increased as waiting times increased (HADS-anxiety: *r* = 0.431, *p* < 0.001; STAI-state anxiety: *r* = 0.219, *p* = 0.001). In the SWR group, waiting times were positively correlated with HADS-anxiety (*r* = 0.587, *p* < 0.001) and STAI-state anxiety scores (*r* = 0.258, *p* = 0.007). Similarly, in the DWR group, a positive correlation was found between waiting times and HADS-Anxiety scores (*r* = 0.261, *p* = 0.006) (Fig. 4). In the SWR group, beta-blocker usage was positively correlated with HADS-anxiety (*r* = 0.331, *p* < 0.001) and STAI-state anxiety scores (*r* = 0.274, *p* = 0.004) (Table 3).

**Table 3:** Correlation analysis between anxiety scale scores and patient characteristics

	SWR group (*n* = 108)				DWR group (*n* = 108)			
	HADS anxiety score		STAI-state anxiety score		HADS anxiety score		STAI-state anxiety score	
	*R*	*p*	*R*	*p*	*R*	*p*	*R*	*p*
Gender	0.163	0.091	0.202	0.036	0.258	0.007	0.154	0.111
Age	−0.056	0.566	−0.039	0.686	−0.149	0.123	0.064	0.509
Pulse rate (per min.)	0.467	< 0.001	0.222	0.021	0.206	0.032	0.109	0.261
Wait time (min)	0.587	< 0.001	0.258	0.007	0.261	0.006	0.154	0.111
Beta blocker use	0.331	< 0.001	0.274	0.004	0.108	0.265	0.084	0.388
Effective dose (mSv)	0.024	0.806	−0.012	0.903	0.123	0.205	0.038	0.696
HADS-depression	0.457	< 0.001	0.306	0.001	0.456	< 0.001	0.373	< 0.001
STAI state anxiety	0.481	< 0.001			0.597	< 0.001		
STAI trait anxiety	0.374	< 0.001	0.533	< 0.001	0.499	< 0.001	0.575	< 0.001

*SWR* standard waiting room, *DWR* designed waiting room, *HADS* hospital anxiety and depression scale, *STAI* state-trait anxiety inventory

In our study, income and education levels had a significant effect on anxiety scores (*p* < 0.05), whereas marital status had no significant impact. Additionally, there was a borderline significant relationship between income and depression scores (*p* = 0.057).

### Scan protocol counts

In the SWR group, Spiral mode was the most commonly performed (75.9%, *n* = 82), followed by adaptive mode (17.6%, *n* = 19) and Flash mode (6.5%, *n* = 7). Similarly, in the DWR group, Spiral mode remained the most prevalent (61.1%, *n* = 66), followed by Adaptive mode (27.8%, *n* = 30) and Flash mode (11.1%, *n* = 12). A significant correlation was found between scan modes and ED in both groups (SWR: *r* = 0.62, DWR: *r* = 0.57, *p* < 0.001). A moderate correlation was observed between BMI and scan modes in both groups (SWR: *r* = 0.22, *p* = 0.022; DWR: *r* = 0.27, *p* = 0.004) (Table 4).

**Table 4:** Relationship between scan protocols, ED (mSv), and BMI

Group	Flash count (%)	Adaptive count (%)	Spiral count (%)	ED correlation	ED *p-*value	BMI correlation	BMI *p-*value
Standard waiting room group	7 (6.5)	19 (17.6)	82 (75.9)	0.62	*p* < 0.001	0.22	*p* = 0.022
Designed waiting room group	12 (11.1)	30 (27.8)	66 (61.1)	0.57	*p* < 0.001	0.27	*p* = 0.004
Total						0.48	*p* < 0.001

*BMI* body mass index

### CCTA image quality assessment

In the analysis of anxiety scores and imaging quality, higher anxiety scores were associated with poorer imaging quality (Spearman correlation coefficient: 0.36, *p* < 0.001). The DWR group demonstrated significantly better imaging quality compared to the SWR group (*p* < 0.001). In the DWR group, imaging quality was rated as excellent in 71 patients (65.7%), whereas this number was 40 (37%) in the SWR group. The proportion of good-quality images was higher in the SWR group, with 39 (36.1%) patients, compared to 31 patients (28.7%) in the DWR group. Additionally, adequate quality was observed in 27 patients (25%), and inadequate quality in 2 patients (1.9%) within the SWR group, while these numbers were 6 (5.6%) and 0 (0%), respectively, in the DWR group. In the comparison between genders, anxiety scores were found to be significantly higher in women than in men (*p* = 0.005); however, no significant difference in imaging quality was observed between genders (*p* > 0.05).

## Discussion

This study highlights the significant benefits of a DWR with music and visual objects on patient outcomes and imaging quality. Patients in the DWR group exhibited lower anxiety levels compared to the SWR group, reflecting the stress-reducing effect of the enhanced environment. These lower anxiety levels were accompanied by reduced pulse rates, indicating a more relaxed physiological state. Consequently, patients in the DWR group required lower doses of radiation, fewer beta-blockers, and achieved better image quality during scans.

The literature includes various studies examining the effects of different music genres on patients’ anxiety levels. Malakoutikhah et al [37], found that pop, Western classical, and Persian traditional music provided relaxation and reduced anxiety in Iranian patients. Other studies on cancer patients have shown that guitar, piano, and flute music positively affect anxiety levels [2, 38, 39]. Based on this literature, we selected music featuring Latin guitar, piano, and flute for the waiting room.

Over the past two decades, studies on music and anxiety in radiology have explored diverse imaging methods, including interventional radiology, CT, ultrasound-guided breast biopsy, mammography, and endorectal prostate ultrasound [12, 40–46]. These studies suggest that music effectively reduces pain and anxiety while improving patient comfort during various procedures.

A study in the literature, similar to our hypothesis but differing in methodology and patient population the effects of waiting room on anxiety, intravenous beta-blocker use, heart rate, and radiation dose in patients undergoing CCTA [12]. No significant effects were observed, likely due to the high initial heart rate in the music group (*p* = 0.017), the inclusion of arrhythmic patients (36%, *n* = 70) undergoing pulmonary vein CT or coronary artery calcium scoring, and the use of nitroglycerin, which may have increased heart rates [12]. Additionally, IV beta-blockers, given to all CCTA patients, equalized heart rates, minimizing radiation dose differences. In contrast, our study utilized three scanning protocols, maintained a routine workflow, and administered oral beta-blockers based on heart rates. The DWR group’s visual and auditory interventions significantly reduced heart rate, anxiety score, radiation dose, and beta-blocker requirements compared to the SWR group.

The literature includes numerous studies examining the effects of music and audiovisual interventions on heart rate and anxiety. Wang et al [47], demonstrated that music reduces pain, respiratory rate, systolic blood pressure, and heart rate during cardiac procedures. Similar benefits have been observed in patients undergoing bronchoscopy [48], myocardial perfusion scintigraphy [49], various imaging procedures [50], breast cancer treatment [51], chemotherapy waiting room [2], and radiotherapy units [52]. Ohana et al [8] found that 74.1% of CCTA patients experience scan-related anxiety. A meta-analysis by Martina de Witte et al [29], confirmed that music therapy reduces heart rate, lowers anxiety, and improves stress-related outcomes. These findings align with our study.

The stress-reducing effects of visual objects are well documented [53–55]. Stationary artwork in waiting rooms has been shown to create a ‘window-like’ effect, reducing anxiety and stress [2, 56]. Visual objects have similarly lowered anxiety in chemotherapy units [2] and emergency department waiting rooms [20]. Beukeboom et al [21] found that artificial nature images reduced anxiety in a radiology department. Similarly, our use of real nature landscapes, like shores and meadows, significantly lowered HADS-anxiety and STAI-state anxiety scores in the DWR group.

Literature highlights a strong link between waiting times and anxiety, with longer waits increasing anxiety in dentistry, radiology, and chemotherapy settings [2, 57, 58]. Prolonged waiting times in the SWR increased HADS-anxiety and STAI-state anxiety scores, while the calming environment in the DWR with music and visual stimuli reduced anxiety and heart rates in CCTA patients.

A chemotherapy unit study [2], found no significant difference in HADS-Depression (*p* = 0.305) or STAI-trait anxiety scores (*p* = 0.535) between groups. Similarly, our study showed no significant differences in these scores (*p* > 0.05), suggesting that visual and auditory interventions may reduce state anxiety but have little effect on depression or trait anxiety.

The mean ED was 11.44 mSv in the SWR group and 9.01 mSv in the DWR group (*p* < 0.001). Compared to Suguru Araki et al [59] our study achieved better doses but higher levels than the PROTECTION VI study [60], and similar or better levels than Yuki Tanabe et al [61]. Menke et al [62], emphasized that prospective ECG-triggered CCTA offers lower doses with comparable quality to retrospective CCTA. Hausleiter et al [60, 63] highlighted dose variability across centers, stressing dose reduction strategies. Our results reflect this variability, and broader use of such strategies may further optimize our doses while preserving diagnostic accuracy.

In dual-source CT studies, heart rate control reduces radiation dose [64, 65]. Beta-blockers are recommended to optimize image quality and reduce radiation, even in third-generation scanners [32, 66]. Our DWR design reduces the need for beta-blockers by creating a calming environment. Langguth et al [67], examined the use of biofeedback before CCTA to reduce heart rate and beta-blocker needs, finding it particularly effective in patients with an initial heart rate of 81–90 bpm. Biofeedback enhances awareness of physical stress signs, helping individuals relax and reduce tension. Similarly, visual and auditory interventions in our DWR group significantly reduced beta-blocker requirements.

In our study, it was observed that anxiety levels decreased as income and education levels increased. This is consistent with the literature, where higher socioeconomic status is associated with better patient knowledge about their condition and easier access to healthcare services [68, 69].

Our study highlights the significant impact of anxiety on imaging quality, consistent with La Grutta et al [14], who reported higher anxiety and reduced image quality in women during CCTA. Unlike their findings, we observed no gender difference, likely due to the anxiety-reducing effects of our DWR interventions. Similarly, Runza et al [35] emphasized heart rate control for optimal image quality through beta-blockers. In our study, the DWR reduced heart rates and beta-blocker use while achieving comparable image quality, supporting the role of non-pharmacological interventions in improving CCTA outcomes.

Our study has some limitations. While auditory exposure duration was measurable, visual exposure duration could not be precisely determined. Social interactions in the waiting room were not recorded, and the effect of mobile phone use on anxiety was not assessed due to ethical constraints. However, a strength of our study is the ease of implementing visual interventions without professional architectural support and the accessibility of the images used.

## Conclusion

This study highlights the effectiveness of visual and auditory enhancements in waiting rooms to reduce anxiety, heart rate, and the need for beta-blockers and radiation before CCTA. By fostering relaxation, these simple, low-cost interventions improve patient cooperation and image quality, offering a practical approach to enhance patient comfort and overall experience in imaging and treatment settings.

### Clinical perspective

Using music and visual stimuli in waiting rooms before CCTA reduces anxiety and heart rate, enhancing image quality, lowering radiation doses, and decreasing beta-blocker use. This approach improves patient comfort and supports more reliable results, offering a practical standard for patient preparation in CCTA.

## Abbreviations

BMI: Body mass index
CAD: Coronary artery disease
CCTA: Coronary CT angiography
CT: Computed tomography
CTDIvol: Computed tomography dose index volume
DLP: Dose-length product
DWR: Designed waiting room
ECG: Electrocardiogram
ED: Effective dose
HADS: Hospital anxiety and depression scale
STAI: State-trait anxiety inventory
SWR: Standard waiting room
